# Rhenium(VII) Compounds as Inorganic Precursors for the Synthesis of Organic Reaction Catalysts

**DOI:** 10.3390/molecules24081451

**Published:** 2019-04-12

**Authors:** Katarzyna Leszczyńska-Sejda, Grzegorz Benke, Joanna Malarz, Mateusz Ciszewski, Dorota Kopyto, Jędrzej Piątek, Michał Drzazga, Patrycja Kowalik, Krzysztof Zemlak, Bartłomiej Kula

**Affiliations:** 1 Hydrometallurgy Department, Instytut Metali Nieżelaznych (IMN), ul. Sowińskiego 5, 44-100 Gliwice, Poland; grzegorzb@imn.gliwice.pl (G.B.); joannam@imn.gliwice.pl (J.M.); mateuszc@imn.gliwice.pl (M.C.); dorotak@imn.gliwice.pl (D.K.); jedrzejp@imn.gliwice.pl (J.P.); michald@imn.gliwice.pl (M.D.); patrycjak@imn.gliwice.pl (P.K.); 2 Syntal Chemicals Sp. z o.o., ul Łabędzka 59. 44-121 Gliwice, Poland; k.zemlak@syntal.com.pl (K.Z.); lab@syntal.com.pl (B.K.)

**Keywords:** rhenium, perrhenates, ammonium complex rhenium(VII) compounds, catalysis

## Abstract

Rhenium is an element that exhibits a broad range of oxidation states. Synthesis paths of selected rhenium compounds in its seventh oxidation state, which are common precursors for organic reaction catalysts, were presented in this paper. Production technologies for copper perrhenate, aluminum perrhenate as well as the ammonia complex of cobalt perrhenate, are thoroughly described. An ion exchange method, based on Al or Cu metal ion sorption and subsequent elution by aqueous perrhenic acid solutions, was used to obtain perrhenates. The produced solutions were neutralized to afford the targeted aluminum perrhenate and copper perrhenate products in high purity. The developed technologies allow one to manage the wastes from the production of these perrhenates as most streams were recycled. Hexaamminecobalt(III) perrhenate was produced by a newly developed method enabling us to produce a high purity compound in a reaction of spent hexaamminecobalt(III) chloride solution with a perrhenic acid. All prepared compounds are the basis for precursor preparation in organic catalysis.

## 1. Introduction

**Figure 1:**
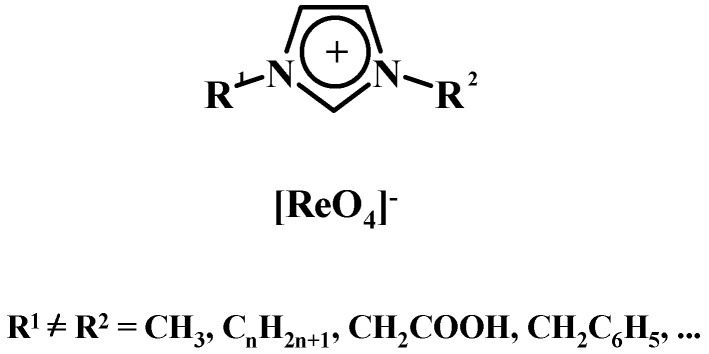
Imidazolium perrhenate.

Rhenium(VII) compounds, like the commonly known organic catalyst methyltrioxorhenium (MTO), may be applied in various catalytic processes as well as used to prepare active catalytic matrices. NH_4_ReO_4_, Al(ReO_4_)_3_ and HReO_4_ are used for the preparation of heterogeneous catalysts of the Re_2_O_7_ type, which is embedded on solid carriers, usually Al_2_O_3_ or more rarely SiO_2_, or another support with a highly developed specific surface area like SiO_2_-Al_2_O_3_, Al_2_O_3_-B_2_O_3_. The most important Re_2_O_7_/Al_2_O_3_ system is characterized by high catalytic activity and selectivity in low a temperature (20–100°C), which is an important advantage. It is used in the metathesis of gaseous and/or liquid alkenes (also these with various functional groups) and acyclic alkenes with functional groups [1,2,3,4,5,6,7,8,9,10,11,12,13,14,15,16,17,18,19]. The Re_2_O_7_/Al_2_O_3_ system was also used in large-scale technologies, e.g., FEAST [20,21] and META-4^®^ [21,22,23]. For a long time it was considered that perrhenates used as catalysts are inactive, e.g., in oxidation reactions. Further research revealed that organic-inorganic compounds that contain a group, or more specifically ReO_4_^−^ anion, are promoters of olefins epoxidation [24,25], sulphide oxidation [26], hydrolysis of cellulose [27] or oxidative esterification of benzaldehydes with alcohols [28]. A major advantage of these catalytic systems compared to metaloorganic catalysts, is their higher stability. They normally constitute a catalytic system component based on ionic liquids, which are formed by an organic part–e.g., imidazole as the ionic liquid (cation) and an inorganic part—a ReO_4_^−^ (anion) from a perrhenate (AgReO_4_, NH_4_ReO_4_) [24,25,26,27,28,29] (Figure 1).

Pyridinium and ammonium perrhenates were also investigated as potential catalysts for the deoxydehydration of diols to afford alkenes [30]. *N*-alkylammonium perrhenate salts [N(R^1^)_3_R^2^]^+^[ReO_4_]^−^ with varying substituents R^1^ (C_4_H_9_, CH_3_ or C_8_H_17_) and R^2^ (C_4_H_9_, CH_3_, C_8_H_17_, C_12_H_25_ or C_16_H_33_) are catalysts in olefin epoxidation processes [31], while [N(hexyl)_4_][ReO_4_] catalyzes the reduction of organic carbonyls and carbon dioxide reactions in the presence of primary and secondary hydrosilanes [32]. All mentioned rhenium(VII) organic salts are usually obtained from easily available ammonium perrhenate and/or perrhenic acid. NH_4_ReO_4_ and NaReO_4_ were also examined as catalysts for the deoxydehydration of glycols in the presence of a reducing agent—sodium sulfite—in organic solvents [33,34] and NH_4_ReO_4_ has found application as a glycol deoxydehydration catalyst with alcohols as reducing agents in high boiling point organic solvents, producing alkene derivatives in high yield [35]. 

Rhenium in the +7 oxidation state has ability to create many complex compounds, however, in this paper only one ammonium complex, i.e., hexaamminecobalt(III) perrhenate was discussed. Ammonium complex compounds containing metals like: copper(II), cobalt(II), cobalt(III), zinc(II), nickel(II), cadmium(II) and have been already described in the literature [36,37,38,39,40]. The history of these compounds dates back to 1933, when synthesis methods and selected properties were reported by Wilke-Dorfurt and Gunzert [37]. One of these compounds was hexaamminecobalt(III) perrhenate produced by the reaction of hexaamminecobalt(III) chloride with an excess of perrhenic acid [37]. Another cobalt complex, namely [Co(NH_3_)_5_(ReO_4_)](ReO_4_)_2_, was described by Lenz and Murmann. Their procedure was composed of two stages: in the first, [Co(NH_3_)_5_H_2_O](ClO_4_)_3_ was dissolved in water and exposed to HReO_4_, resulting in the formation of [Co(NH_3_)_5_(H_2_O)](ReO_4_)_3_·2H_2_O, which in the second stage was dried initially under vacuum for 3 h at 50–60 °C to produce a pink [Co(NH_3_)_5_(H_2_O)](ReO_4_)_3_ complex, followed by further drying at 95–130 °C for 2–5 h to obtain the violet [Co(NH_3_)_5_(ReO_4_)](ReO_4_)_2_ [38].

In the literature syntheses of tetraamine complexes of zinc and cadmium can be found as well. These are obtained by adding concentrated ammonia solutions to suitable metal perrhenates [37,39]. Thermal gravimetric analysis (TGA) for both complexes indicated the loss of ammonia units with the increase in temperature, which can be ascribed to the formation of [M(NH_3_)_2_](ReO_4_)_2_ and M(ReO_4_)_2_ (where M is: Zn, Cd). Zinc(II)-diamine perrhenate is formed in the 150–95 °C temperature range, while anhydrous zinc(II) perrhenate is formed at 230–275 °C. For the formation of cadmium(II)-diamine perrhenate and cadmium(II) perrhenate the temperature ranges are 100–150 °C and 180–230 °C, respectively [39]. 

It was found using Raman spectroscopy that the ReO_4_^−^ anion is present in ionic form and is not coordinated with the metal atom, while X-ray diffraction showed the isostructurality of the following compounds: [Zn(NH_3_)_4_](ReO_4_)_2_, [Cd(NH_3_)_4_](ReO_4_)_2_ and [Co(NH_3_)_4_](ReO_4_)_2_ [39]. 

Preparation procedures of perrhenates described in the literature do not however pay enough attention to their environmental and economic aspects. Rhenium is a rare and valuable metal, with an annual output of ca. 60 tonnes. Application of suitable auxiliary operations could allow minimization of rhenium losses and maximize the purity of the compounds. 

This publication presents production methods for aluminum and copper(II) perrhenates, and the ammonia complex of cobalt(III) perrhenate. These compounds are precursors for catalytic system preparation or are constituents of such systems. All the described technologies are complete, including management of generated wastes, and could be easily applied in industry. They were scaled up and tested so it is possible to implement them even in a continuous production line. Currently, these technologies constitute an offer made by the industrial partner Syntal Sp.z.o.o (Gliwice, Poland) for the Institute of Non-Ferrous Metals (IMN, Gliwice, Poland). 

## 2. Results and Discussion

### 2.1. Perrhenates of Various Metals–Ion-Exchange Method

#### 2.1.1. Copper(II) Perrhenate

Ten cycles using the selected ionite–C160 in hydrogenated form–were performed. Then the ionite was regenerated for the next five cycles. In each cycle 8 dm^3^ of nitrate solution containing 5 g/dm^3^ Cu was used. Table 1 presents the copper ion sorption results for the first ten cycles, while Table 2 shows the data for the remaining five cycles (after regeneration with nitric acid). 

**Figure 2:**
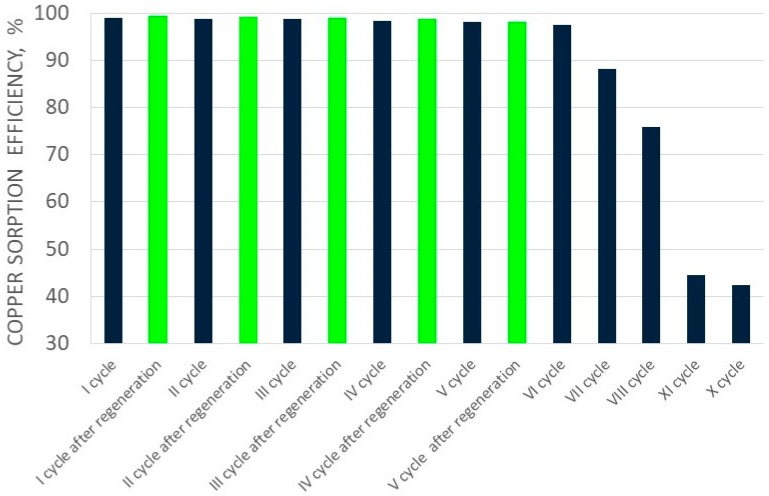
Changes in copper sorption efficiency in subsequent cycles (I-XV).

**Figure 3:**
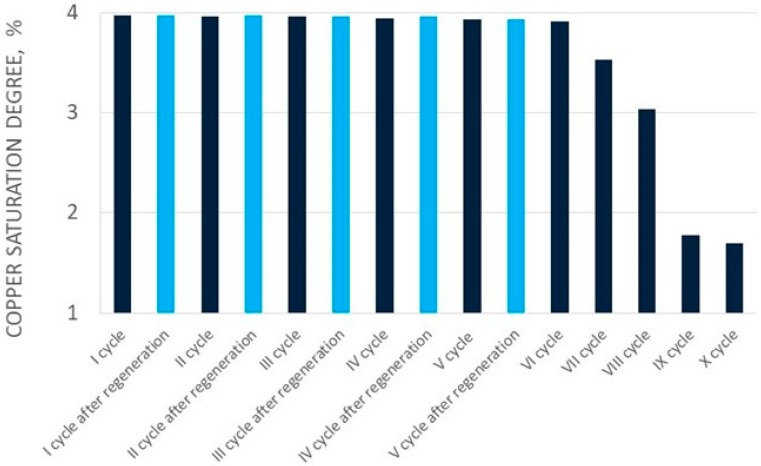
Changes in copper saturation degree in subsequent cycles (I-XV).

**Figure 4:**
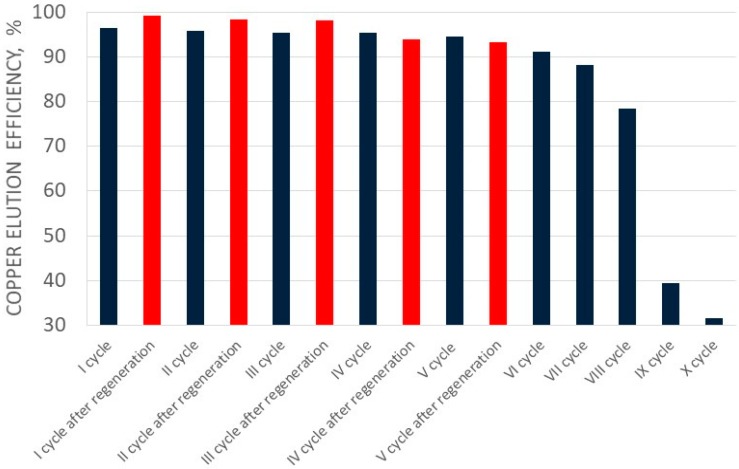
Changes in copper elution efficiency in subsequent cycles (I-XV).

Figure 2 and Figure 3 show the change of copper(II) ions sorption efficiency, and the ionite saturation ratio with copper(II) ions in fifteen cycles, respectively. Elution of sorbed copper(II) ions in the first cycles was conducted with 3.0 dm^3^ of perrhenic acid containing 80.0 g/dm^3^ Re. In the next cycles the solution of perrhenic acid of 120.0 g/dm^3^ Re mixed with first part of the solution after elution was used. Addition of HNO_3_ and H_2_O_2_ in each cycle was constant. Elution results for copper(II) ions for the first ten cycles are shown in Table 3, and for the last five cycles (after regeneration with nitric acid) in Table 4. Figure 4 presents the change of elution efficiency of copper(II) ions in fifteen cycles.

Sorption efficiency in the first five cycles was very high—over 97.0%—therefore the ionite saturation ratio was correct–above 3.0% for the first eight cycles. It is important that the copper concentration in solution resulting from sorption was low—from 0.04 to 0.10 g/dm^3^ for the first six cycles. This means that the copper losses in this stage of the process were minimal. It has to be mentioned that copper sorption efficiency in each of the ten cycles was slightly decreasing up to cycle VIII. Between cycles VIII and IX the sorption efficiency of copper decreased significantly to about 30%.

Elution efficiency in the first six cycles was high–over 91% with a decreasing tendency. The efficiency ranging from the first to the eighth cycle from 99.13 to 75.93%, respectively, and dropped in cycle IX to 44%. It can be stated that C160 resin worked properly under the selected conditions only for eight cycles with appropriate sorption and elution parameters, and after that the resin should be regenerated.

After the cycle X assumptions were achieved (i.e., reduction of ionite saturation to less than 2% in the next two cycles, increase in copper concentration to 2.60 g/dm^3^, and reduction of sorption efficiency to about 40%), and resin was sent for regeneration. Copper regeneration was carried out at a maximum efficiency over 99%, using 1 dm^3^ 32% nitric acid solution. After regeneration and washing of the acid from the ionite, the total solution volume was 2.6 dm^3^, containing 4.4.g/dm^3^ Cu. This was directed to the extraction of crude copper(II) nitrate. The subsequent cycles using regenerated resin confirmed the effectiveness of the regeneration. The sorption efficiency of copper ions was over 98%, which was comparable with values before regeneration. The degree of saturation of Cu^2+^ ionite in subsequent cycles after its regeneration ranged from 3.97 to 3.93%. The elution efficiency was in a range from 93.28 to 99.34%. It was found that the selected resin under cyclic conditions was featured by appropriately high sorption and elution parameters to the next ionite cycle VIII, and then the resin should be regenerated and directed to sorption of copper(II) ions. 

Solutions after elution were neutralized and the results are presented in Table 5, while the composition of obtained copper(II) perrhenate before and after purification is shown in Table 6. It is possible to precipitate copper(II) perrhenate with the standard composition in a reproducible way from the neutralized solution. The elevated temperature significantly increased the neutralization reaction rate, both using copper(II) oxide and copper(II) hydroxide. It was therefore considered that neutralization should be carried out at 80°C, using commercial copper(II) oxide as the neutralizing agent. The use of hydroxide requires its continuous precipitation, which is cumbersome under industrial conditions.

**Figure 5:**
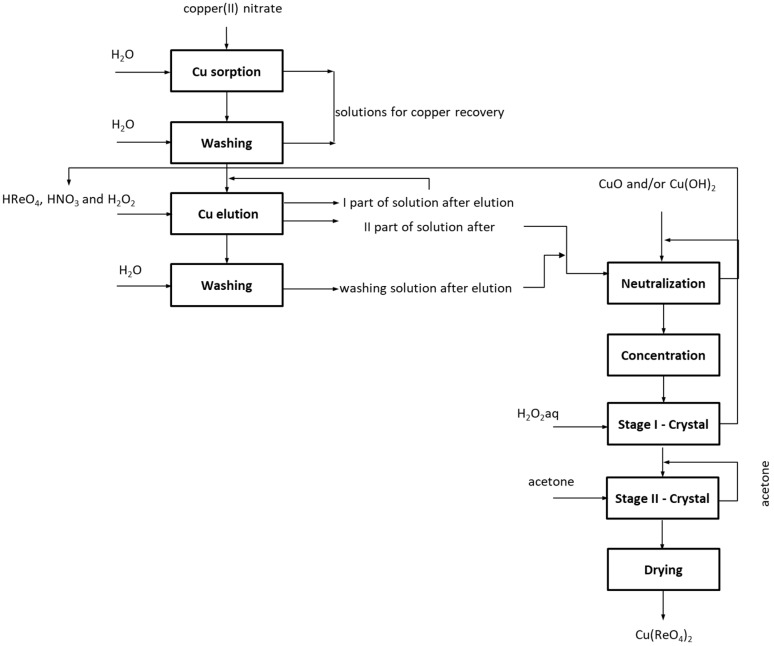
Scheme of the technology for copper(II) perrhenate production.

**Figure 6:**
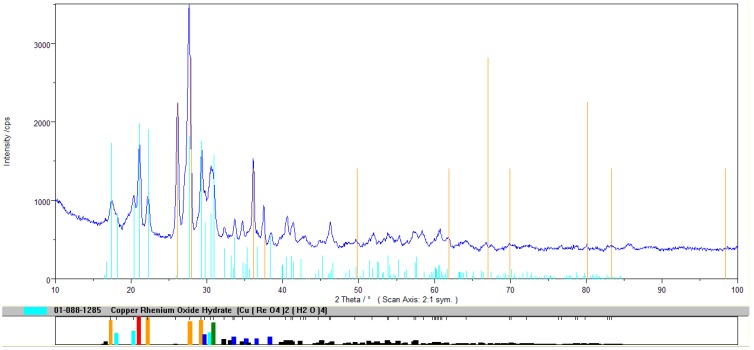
XRD pattern for precipitate copper(II) perrhenate.

**Figure 7:**
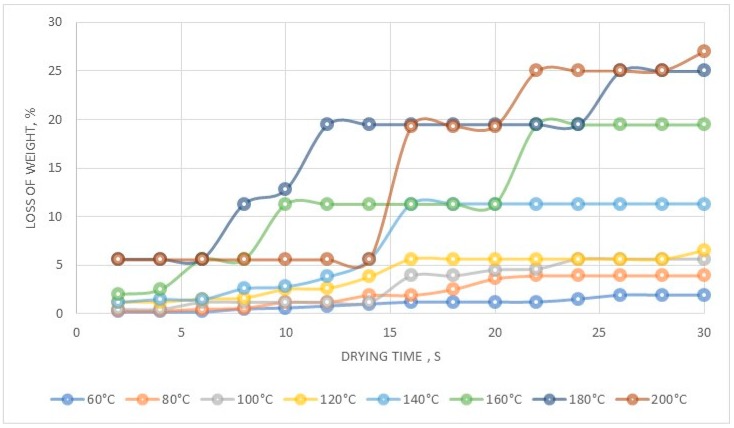
Thermal stability of the precipitated Cu(ReO_4_)_2_.

**Figure 8:**
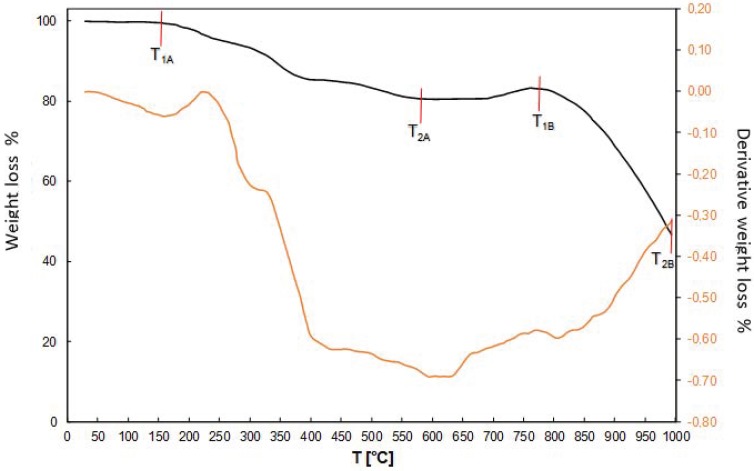
Thermal stability of the anhydrous Cu(ReO_4_)_2_.

The crude compound was purified from nickel and cobalt, using a 15% solution of hydrogen peroxide, while the use of acetone allowed us to obtain a compound of high purity. It has also been proven that the resin after regeneration was suitable for the production of high purity copper(II) perrhenate. These experiments allowed us to develop thr technological scheme presented in Figure 5. The XRD pattern of the precipitated compound in a hydrated form is shown in Figure 6, while its thermal stability is shown in Figure 7. DTA/TG analysis of the anhydrous compound is presented in Figure 8.

It can be noticed that at 140°C it is possible to produce anhydrous copper perrhenate while a further increase in temperature leads to its decomposition. A systematic and ongoing decomposition started at 159 °C.

#### 2.1.2. Aluminum Perrhenate

**Figure 9:**
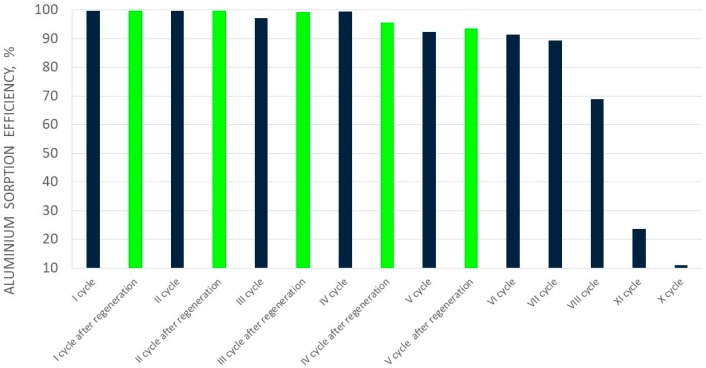
Changes in aluminium sorption efficiency in subsequent cycles (I-XV).

**Figure 10:**
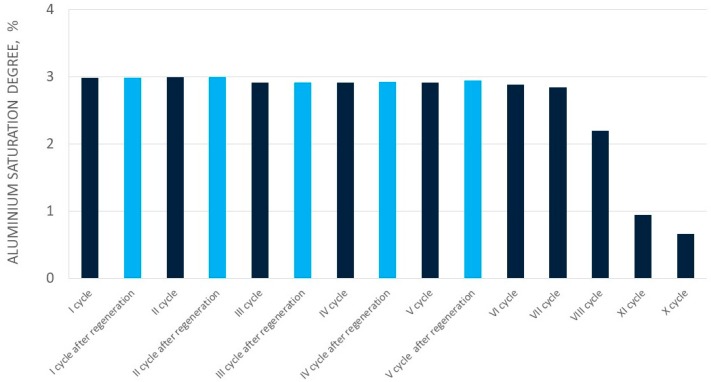
Changes in aluminium saturation degree in subsequent cycles (I-XV).

**Figure 11:**
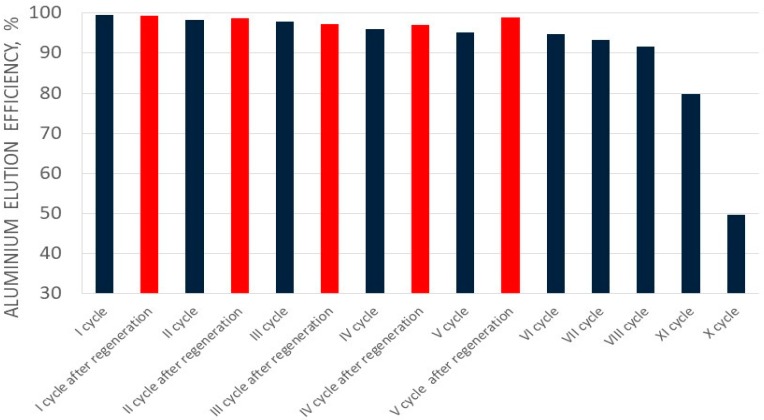
Changes in aluminium elution efficiency of the ionite in subsequent cycles (I-XV).

In the case of aluminum perrhenate, ten cycles of selected ionite—PFC 100x10 in the hydrogenated form—were examined. Then the ionite was regenerated and five more cycles were tested. In each cycle, 5.0 dm^3^ of an aqueous nitrate solution containing 5.0 g/dm^3^ of Al was passed through the ionite bed. The results of aluminum ion sorption tests for the first ten cycles, and for the next five (after regeneration with nitric acid), are presented in Table 7 and Table 8, respectively. Figure 9 and Figure 10 show the change in the sorption efficiency of aluminum ions and the degree of saturation of aluminum ionite in fifteen cycles, respectively. In Table 9 and Table 10 the results for the elution process were collected, with the division of aluminum elution efficiency into two products, i.e., product A (main–aluminum perrhenate) and product B (a by-product: aluminum oxide with rhenium). The change in elution efficiency of aluminum ions in subsequent cycles (ten before regeneration and five after regeneration) can be seen in Figure 11.

The sorption efficiency in the first six cycles was very high and exceeded 90%, hence the saturation degree of the ionite was correct and reached around 3% for the first seven cycles. The concentration of aluminium in the solutions formed after sorption ranged from 0.08 to 0.23 g/dm^3^ for the first seven cycles. These solutions were used to prepare another solution for sorption in the next ionite work cycle. It should be noted that aluminum sorption efficiency in all ten cycles decrease slightly until cycle VII. Between cycles VIII and X, the aluminum sorption efficiency decreased significantly–over 60%.

The elution efficiency of aluminum ions in the first eight cycles was high and exceeded 90%, but a decreasing trend should be mentioned. Until cycle VIII, the elution efficiency was 91.71%, then it dropped significantly to 50% in cycle X. Thus, it can be concluded that PFC 100 × 10 resin worked properly under specific conditions, achieving appropriate sorption and elution parameters until cycle VIII and it is recommended to then regenerate it.

After ten cycles the planned assumptions were achieved (reduction of the ionite saturation to less than 2% in two subsequent cycles, increase in aluminum concentration, and decrease in sorption efficiency to about 11%), and the resin was sent to regeneration. Aluminum regeneration was carried out at a maximum efficiency over 99%, using 1 dm^3^ 32% nitric acid solution. After regeneration and washing of the acid from the ionite, a total volume of solution of 2.1 dm^3^ containing 3.2 g/dm^3^ Al was obtained, which was further sent to the extraction of crude aluminum nitrate.

Subsequent cycles performed using regenerated resin confirmed the effectiveness of the regeneration. The sorption efficiency of aluminum ions was equally high and sometimes even higher and more stable than before regeneration, i.e., over 93%. The saturation degree of the Al^3+^ ionite after its regeneration, in the subsequent cycles was stable–around 3%. The elution efficiency was over 93% in all examined cycles after regeneration. It was found that the selected resin under recycling conditions features appropriately high sorption and elution parameters until the next eight cycles. Then the resin should be regenerated and subsequently directed to aluminum ion sorption.

**Figure 12:**
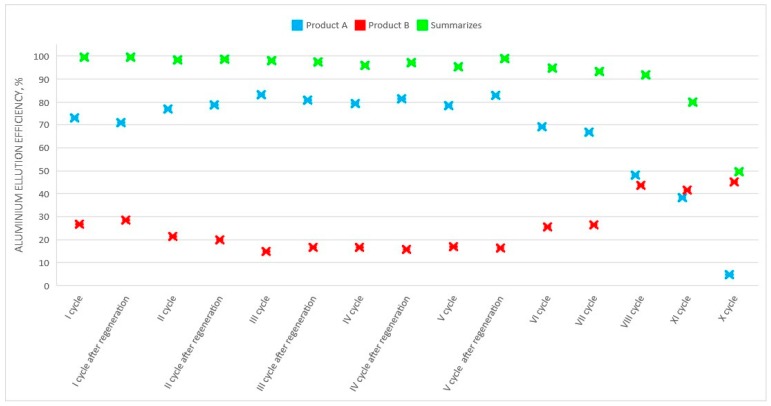
Changes in elution efficiency of the ionite by Al in subsequent cycles (I-XV) for two products (A and B).

**Figure 13:**
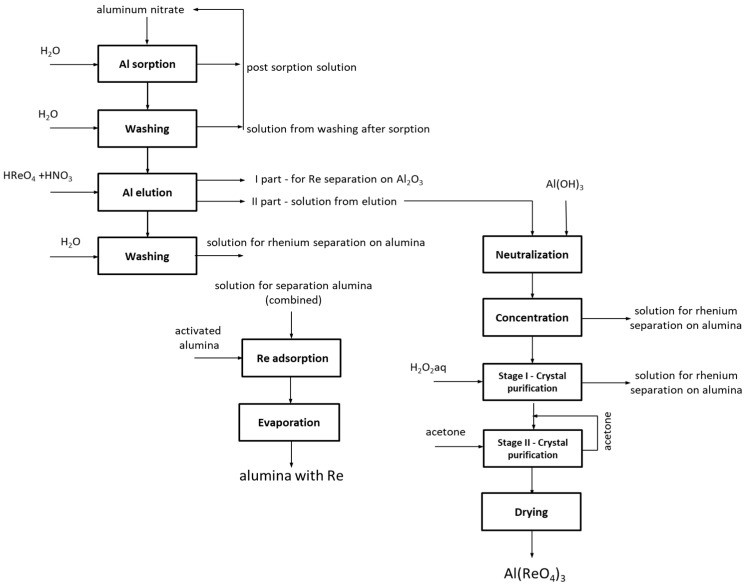
Scheme of the technology to produce aluminum perrhenate.

An important element of this technology is the division of eluate and water after elution into the target product A (aluminum perrhenate) and product B (aluminum oxide with rhenium). It can be clearly seen in Figure 12 that in the first cycles there are significantly more solutions, which meet the requirements for the production of product A, however, and this value decreases in subsequent cycles. The amount of aluminum for the extraction of product A, at stage VIII reached the same level of aluminum directed to the production of product B. Then the number of solutions for the extraction of product B started to increase, see Figure 13.

The solutions obtained after elution and washing after elution were used in the neutralization tests. Some were used to extract product A and others product B. The obtained results are presented in Table 11 and Table 12. The composition and purity of the obtained components are illustrated in Table 13.

The results showed that an increase in temperature significantly enhanced the eluate neutralization with aluminum hydroxide. At 80 °C, it is possible to neutralize the solution after 1.5 h, which is then filtered, concentrated, purified and dried. High purity aluminum perrhenate with a stoichiometric composition (Table 13) can be obtained. However, in case of product B Al_2_O_3_ was used as neutralizing agent. Different temperature and amount of neutralizing agent, may lead to product B containing from 73.2 to 79.6% R, affording a good precursor for the production of heterogeneous catalysts. The performed experiments allowed to present the scheme of the developed technology–Figure 13. 

**Figure 14:**
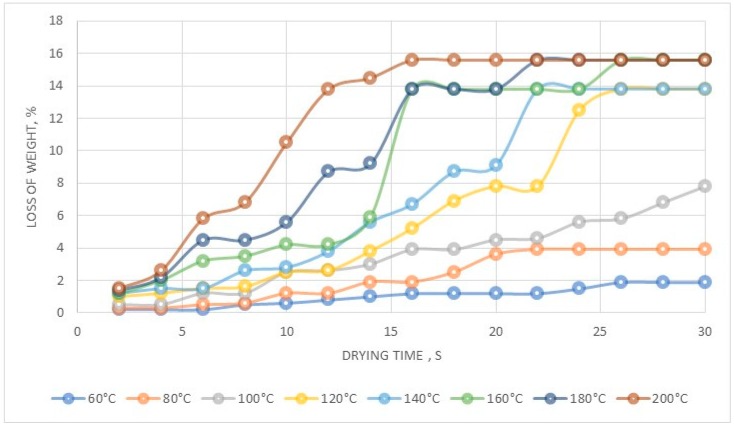
Thermal stability of the precipitated Al(ReO_4_)_3_.

The thermal stability is shown in Figure 14. Evaporation allowed us to produce octahydrated aluminium perrhenate (Figure 14). Drying at 120–160 °C gave aluminium perrhenate monohydrate while at 160–200 °C the anhydrous form was obtained. 

### 2.2. Ammoniacal Rhenium(VII) Complexes

#### Hexaamminecobalt(III) Perrhenate

**Figure 15:**
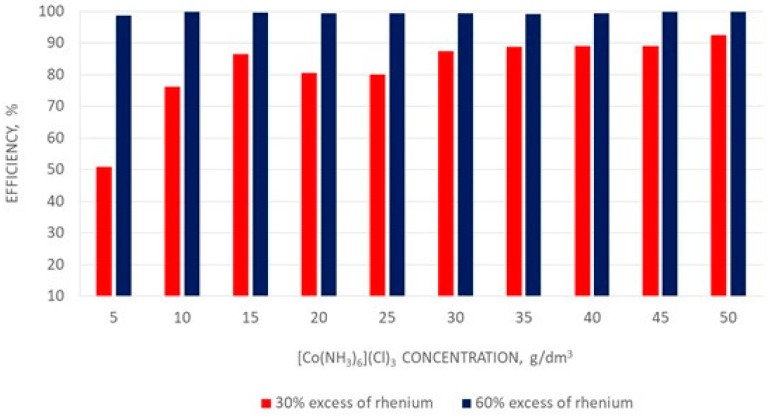
Effect of [Co(NH_3_)_6_]Cl_3_ concentration on the precipitation efficiency of hexaamminecobalt(III) perrhenate.

At the beginning the effect of hexaamminecobalt(III) chloride concentration on the precipitation efficiency of hexaamminecobalt(III) perrhenate was determined. The tests were carried out using 30% and 60% excess of rhenium with respect to cobalt–tests results are presented in Figure 15.

It was observed that the increase in [Co(NH_3_)_6_]Cl_3_ concentration in solution enhanced the precipitation efficiency of [Co(NH_3_)_6_](ReO_4_)_3_·2H_2_O, when 30% excess of rhenium to cobalt was used, while for 60% excess rhenium, the precipitation efficiency in the entire examined range of [Co(NH_3_)_6_]Cl_3_ concentration, was very high and over 99%. It was found that a high concentration of hexaamminecobalt(III) chloride promoted high precipitation efficiency, but was not a sufficient condition. Intentional low cobalt concentration (less than 0.002 g/dm^3^ Co) in the solutions remaining after precipitation [Co(NH_3_)_6_](ReO_4_)_3_·2H_2_O was obtained only with an 60% excess of perrhenic acid. Further investigations were carried out with a solution of hexaamminecobalt(III) chloride at a concentration of 30 g/dm^3^. The effect of temperature in the range from 10 to 80 °C was investigated too. The results of these tests are presented in Table 14.

It was found that the temperature did not affect the precipitation efficiency of hexaamminecobalt(III) perrhenate. In the entire temperature range, the ammonia complex of cobalt perrhenate precipitation efficiency was over 90%. Regardless of the applied temperature, [Co(NH_3_)_6_](ReO_4_)_3_·2H_2_O with the stochiometric composition was obtained. Results concerning the reaction time influence on process efficiency are presented in Table 15.

**Figure 16:**
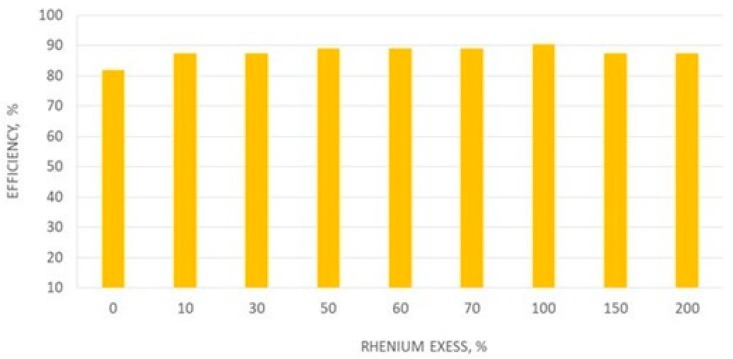
Effect of the rhenium excess used (in the form of HReO_4_) on the precipitation efficiency [Co(NH_3_)_6_](ReO_4_)_3_·2H_2_O.

There was no effect of reaction time on the precipitation efficiency of hexaamminecobalt(III) perrhenate dihydrate. The precipitation efficiency was above 90% in the entire tested range. Therefore, it was recommended to use half-hour reaction time in subsequent studies. The effect of rhenium excess was also examined, and results are presented in Figure 16.

The obtained precipitation efficiency [Co(NH_3_)_6_](ReO_4_)_3_·2H_2_O was high in all cases, regardless of the amount of perrhenic acid used. In each case [Co(NH_3_)_6_](ReO_4_)_3_·2H_2_O was obtained with the stochiometric composition. However, the use of 50% excess of acid, allowed us to obtain waste solutions containing less than 0.002 g/dm^3^ Co. Results for the effect of perrhenic acid concentration on the precipitation efficiency and composition of [Co(NH_3_)_6_](ReO_4_)_3_·2H_2_O are presented in Table 16.

It was found that the increase in perrhenic acid concentration enhanced the precipitation efficiency of [Co(NH_3_)_6_](ReO_4_)_3_·2H_2_O. It was about 90% for acids with rhenium concentrations above 100 g/dm^3^ Re, while for rhenium concentrations below 100 g/dm^3^ Re, the precipitation efficiency of [Co(NH_3_)_6_](ReO_4_)_3_·2H_2_O gradually decreased. For perrhenic acid with rhenium concentration 16 g/dm^3^ Re, the precipitation efficiency was less than 50%. Therefore, it was found that for precipitation of [Co(NH_3_)_6_](ReO_4_)_3_·2H_2_O, perrhenic acid with concentration of rhenium above 100 g/dm^3^ should be used.

Precipitation of hexaamminecobalt(III) perrhenate dehydrate was additionally performed using various precipitating agents i.e.,: ammonium perrhenate, and anhydrous cobalt(II) perrhenate. The results obtained are presented in Table 17.

It was found that use of ammonium perrhenate solution with a maximum rhenium concentration of 20 g/dm^3^ (at room temperature) was not sufficient to precipitate all of the cobalt from solution, therefore this procedure was discarded. At 60% excess of rhenium, about 50% of the cobalt remained in the solution. The use of solid ammonium perrhenate eliminated the problem of limited rhenium concentration in aqueous solutions of this salt. However, it was noticed that the use of this agent in solid form caused co-precipitation of NH_4_ReO_4_ with [Co(NH_3_)_6_](ReO_4_)_3_·2H_2_O. The use of anhydrous cobalt(II) perrhenate as the precipitation agent caused the precipitation of a flesh-colored substance. This was identified by X-ray and quantitative analysis that confirmed basic component of the tested sample in form of [Co(NH_3_)_6_](ReO_4_)_3_·2H_2_O significantly contaminated with cobalt(II) perrhenate and [Co(NH_3_)_6_]Cl_3_. After the analysis it was concluded that none of the proposed compounds could replace perrhenic acid for the preparation of [Co(NH_3_)_6_](ReO_4_)_3_·2H_2_O].

The subsequent tests enabled us to verify whether the commercial hexaamminecobalt(III) chloride [Co(NH_3_)_6_]Cl_3_ can be replaced by product obtained within this research, as well as if is it possible to precipitate the hexaamminecobalt(III) perrhenate dihydrate from the hexaamminecobalt(III) chloride solution produced after separation of activated carbon in the synthesis of [Co(NH_3_)_6_]Cl_3_ used i the crystallization of hexaamminecobalt(III) chloride. The results obtained were presented in Table 18.

Using the obtained [Co(NH_3_)_6_]Cl_3_ and ammonia solutions containing Co, hexaamminecobalt(III) perrhenate dihydrate was produced with a high efficiency of about 96%, and with a stoichiometric composition. The use of ammonia solutions is more convenient as it allows the reduction of the number of unit operations, i.e., crystallization and recrystallization of [Co(NH_3_)_6_](ReO_4_)_3_·2H_2_O.

**Figure 17:**
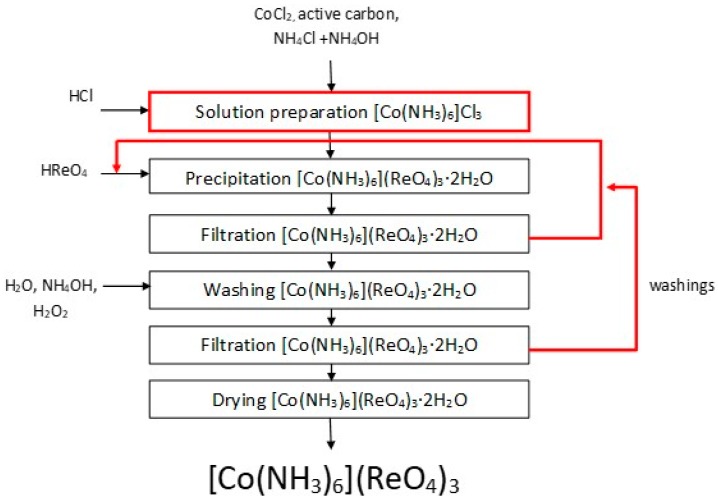
Scheme of the technology to produce [Co(NH_3_)_6_](ReO_4_)_3_.

**Figure 18:**
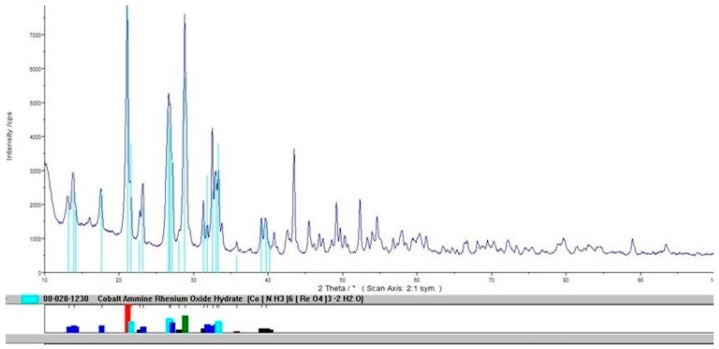
XRD pattern of precipitated [Co(NH_3_)_6_](ReO_4_)_3_·2H_2_O.

**Figure 19:**
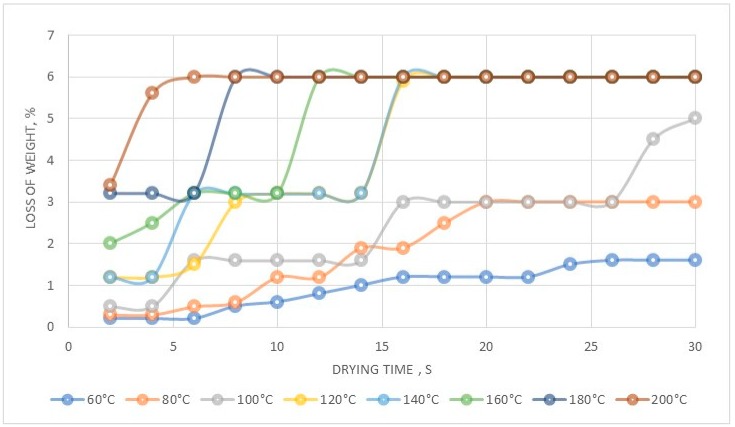
Thermal stability of precipitated [Co(NH_3_)_6_](ReO_4_)_3_.

The performed experiments allowed us to develop a new method to produce anhydrous hexaamminecobalt(III) perrhenate—dried at 120°C—with a high efficiency over 90%. The obtained compound was quantitatively analyzed, to confirm preparation of [Co(NH_3_)_6_](ReO_4_)_3_ with the following composition: 61.3% Re, 6.5% Co, 11.2% NH_3_, <20 ppm Cl, <5 ppm Na, <5 ppm Ca, <5 ppm Mg, <5 ppm Pb, <5 ppm Fe, <5 ppm Mo, <5 ppm Cr, <5 ppm Ni, <10 ppm K, <3 ppm Zn. The technological scheme for this process is shown in Figure 17. The XRD pattern of the precipitated compound in a hydrated form is shown in a Figure 18, while its thermal stability is presented in Figure 19.

It was possible to produce dihydrate hexaamminecobalt(III) perrhenate (Figure 19). Then, drying above 120 °C allowed to produce the anhydrous form of this compound that was stable in the broad temperature range.

## 3. Experimental Section

### 3.1. Materials

Perrhenic acid of high purity, produced at the IMN using extraction as well as ion-exchange method (rhenium concentrations above 300.0 g/dm^3^), was used as the source of rhenium in all experiments. Perrhenic acid contained 50-900g/dm^3^ Re and <20 ppm ammonium ions, <2 ppm As; <2 ppm Bi, <3 ppm Zn, <3 ppm Mg, <3 ppm Cu, <5 ppm Mo, <5 ppm Ni, <5 ppm Pb, <10 ppm K, <3 ppm Ca, <3 ppm Fe, <3 ppm Al, <5 ppm Co [41,42,43,44]. High purity nitric acid (HNO_3_) and 30% solution of hydrogen peroxide (POCH, Gliwice, Poland) were also used. Cobalt complex was synthesized from ammonium perrhenate (KGHM Polska Miedź S.A., Głogów, Poland) and cobalt(II) perrhenate (produced based on technology developed at IMN) [45,46,47,48]. In ion-exchange experiments two acidic resins, namely, C160 and PFC 100 × 10 (Purolite, Gdańsk, Poland) were used. In the ammonium complex production, [Co(NH_3_)_6_]Cl_3_ (Acros Organics, Trenton, NJ, USA) was used. Additionally, aluminum, copper and cobalt salts and oxides (Acros Organics) were also used. Purification was performed using anhydrous extra purity acetone (POCH, Gliwice, Poland).

### 3.2. Various Metals Perrhenates–Ion-Exchange Methods

Ion-exchange method was used for nickel(II), cobalt(II), cesium, rubidium, copper(II), chromium(III) and aluminum perrhenates production, where sorption of nickel, cobalt, cesium, rubidium, copper, chromium and aluminum cations occurred. In the next step, these cations were eluted with aqueous solutions of perrhenic acid. The production method of perrhenic acid depends on the planned further use of the mentioned perrhenates. It can be obtained either by ion-exchange method (for superalloy precursors production) [41,42,43] or by solvent extraction [44] (for catalyst preparation). Solutions from the elution step were mixed with the other one, e.g., obtained from the washing after elution, and directed for the evaporation (in case of Ni, Co, Cr, Cs, Rb, Al, Cu) or crystallization (in case of *nano*-Rb, *nano*-Cs). Produced perrhenates were washed and dried to obtain their final form. Production methods of perrhenates of selected metals, i.e., nickel(II), cobalt(II), chromium(III) were presented in details in previous publications of the main author [45,46,47,48,49,50,51]. It has to be mentioned that the important techno-economical factor of these technologies is attributed to the applied recycles that influence product purity and technology efficiency. The production methodologies of copper(II) perrhenate and aluminum perrhenate were described.

Parameters for sorption process were: sorption efficiency (described as the mass ratio of Cu or Al sorbed in ionite to their mass in initial solutions times 100%), and ionite saturation ratio (described as mass ratio of Cu or Al in the solution directed to neutralization to mass of the components sorbed in ionite times 100%). Elution efficiency was calculated as the ratio of metal mass in the solution before neutralization to metal mass sorbed in the ionite, multiplied by 100%.

#### 3.2.1. Copper(II) Perrhenate

The first preparation of copper(II) perrhenate hydrates from copper(II) carbonate and perrhenic acid was reported in 1931 by Briscoe et al. [36]. In this paper synthesis of copper(II) perrhenate using copper-containing aqueous nitrate solutions was described. Research conducted in dynamic and static conditions allowed us to identify C160 ionite in the hydrogenated state as the most effective resin. Preliminary conditions for each process step were chosen during experiments too. Consequently, it was assumed that the ionite saturation ratio required to conduct process properly needs to be between 2.0 and 4.0%. It was considered that the sorption of copper(II) ions should be performed in such way that the final concentration of copper in the solution does not exceed 0.5g/dm^3^. Research on a large-laboratory scale under dynamic conditions were performed with 1.0 kg of the ionite placed inside an ion-exchange column. The ratio of the column’s height to its diameter was above 8.5. Due to the cyclic character of the process and ability to verify the results only after conducting few cycles it was decided to do ten cycles. Experiments were conducted until the ionite saturation ratio with copper ions was 2.0% in two consecutive cycles. Ionite after cycle X was regenerated with 32% nitric acid solution and tested for another five cycles. Sorption was performed using a solution containing 5.0 g/dm^3^ Cu from the top of the column, at room temperature with the flow rate of 5.0 dm^3^/h. Ionite after the sorption was washed with water from the top of the column, using about 1.5 dm^3^ of water on each 1 kg of ionite, with a flow rate of 7.5 dm^3^/h. Solutions resulting from washing were mixed with that from sorption, and managed. Washed resin was eluted with aqueous solution of perrhenic acid 80–120 g/dm^3^ Re, using above 6.0 g of rhenium on each 1 g of copper absorbed in the ionite, with addition of 0.05–0.10 dm^3^ concentrated nitric acid and 0.025–0.050 dm^3^ 30% solution of hydrogen peroxide on each 1 dm^3^ of eluting agent (perrhenic acid). Elution was performed from the top to the bottom of the column with a flow rate of 2.5 dm^3^/h. Solutions from the elution were stored in two portions–1/3 of the total volume, which was mixed with solutions resulting from purification, was recycled to the eluting agent preparation step, and 2/3 of the total volume was mixed with solutions resulting from washing after elution and directed to neutralization. The eluted ionite bed was washed with water using about 3.0 dm^3^ of water for each 1 kg of ionite with flow rate of 3.0–4.0 dm^3^/h. Solutions from washing after elution were collected and then mixed with qcsecond portion of solution resulting from elution. Such a mixture was treated with copper(II) oxide and/or copper(II) hydroxide in order to obtain a solution of stoichiometric composition of copper to rhenium, which is favorable for extraction of the final product–copper(II) perrhenate. Solution neutralization was performed at 80 °C until the pH between 3.5–7.2 was achieved. Post-neutralization solution was filtered at elevated temperature to remove solid impurities. Concentrating of the neutralized solution was performed at temperature up to 80 °C with vigorous mixing until completely dried. Three temperatures, i.e., 20, 60 and 80 °C and two neutralizing agents (copper(II) oxide and freshly precipitated copper(II) hydroxide) were tested. During neutralization and concentration steps, 30% solution of hydrogen peroxide was added to prevent the reduction of copper(II) ions. Obtained precipitates were dried in air, and then purified in two steps: firstly with 15% aqueous solution of hydrogen peroxide at ≤5 °C (using 0.002 dm^3^ of agent on each 1.0 g of precipitate) and then anhydrous acetone washing at ≤10 °C (using 0.0005 dm^3^ of acetone per each 1.0 g of precipitate). Aqueous solution from the washing was mixed with the first portion of solution resulting from elution and then recycled to eluting agent preparation step. Same portion of acetone was used in all fifteen cycles. Precipitate after purification was dried at the temperature below 140 °C until constant mass was obtained. The obtained anhydrous copper(II) perrhenate with stoichiometric composition was then analyzed for the content of selected impurities.

#### 3.2.2. Aluminum Perrhenate

The history of Al(ReO_4_)_3_ dates back to 1968 when it was first synthesized by Varfolomeev et al. [52]. Since then this compound is produced in aluminum salt (either nitrate or carbonate) reactions with perrhenic acid. The production of anhydrous aluminum perrhenate, which was described in this publication, was conducted with an aqueous solution of aluminum nitrate as the metal source. Based on results from dynamic and static conditions on various scales PFC 100 × 10 in hydrogenated form was the most effective resin. In these experiments preliminary parameters of each operations were chosen. It was assumed that the ionite saturation ratio must be above 2.0% for the process to proceed properly. It was considered that the sorption of aluminum ions should be continued to the final aluminum concentration below 1.0 g/dm^3^. Large-laboratory scale research was conducted under dynamic conditions using an ion-exchange column, with a height to diameter ratio above 10. The column was loaded with 1 kg of ionite. Due to the cyclical nature of the process and ability to verify results only after performing a few cycles ten cycles of ion-exchange were done. Research was conducted until the ionite saturation ratio with aluminum ions was below 1.0% in two consecutive cycles. Ionite after cycle X was regenerated with 32% nitric acid solution and tested for another five cycles. Sorption was performed using solution containing 5.0 g/dm^3^ Al, from the top to the bottom of the column, at room temperature with a flow rate of 3.0 dm^3^/h. After sorption the ionite was washed with water from the top to the bottom of the column, using about 1.5 dm^3^ of water on each 1.0 kg of ionite with a flow rate of 3.0 dm^3^/h. Solutions resulting from washing after sorption were mixed with solutions resulting from sorption and used to dissolve aluminum nitrate. Washed resin was eluted with aqueous solution of perrhenic acid of 200 g/dm^3^ Re concentration, using 21.0 g of rhenium on each 1.0 g of aluminum, with addition of 0.15 dm^3^ nitric acid on each 1.0 dm^3^ of eluting agent (perrhenic acid). Elution was performed from the top to the bottom with a flow rate of 1.5 dm^3^/h. Solutions resulting from elution were collected in two portions: first one-bed volume was mixed with solution resulting from washing after elution, while the second 2-5 bed volumes (BV) was neutralized and crystallized. Eluted ionite bed was washed using 2.0–5.0 dm^3^ of water on each 1 dm^3^ of ionite with a flow rate of 3.0 dm^3^/h. Solutions obtained from washing after elution were collected and mixed with the first portion of solution from elution and then sent to rhenium extraction on aluminum oxide. The second portion from elution (addressed to neutralization, concentration and separation), which contained an excess of rhenium vs aluminum, was treated with freshly precipitated aluminum hydroxide in a stoichiometric amount to the excess of rhenium in the solution. It was fed as aqueous solution washed from ammonium ions to the level <0.1%. This operation was performed at the temperature below 80 °C until all the aluminum oxide reacted, i.e., until a pH in the range from 3.5 to 4.4 was obtained. After neutralization, the solution was filtered at elevated temperatures to remove solid impurities. The solution was neutralized and concentrated at a temperature below 80 °C with intensive mixing until completely dry. Obtained precipitate was purified in two steps: washing with demineralized water at temperature ≤10 °C (using 0.001–0.020 dm^3^ of water on each 1 g of precipitate), and then with anhydrous acetone at temperature ≤10 °C (using 0.0005–0.0050 dm^3^ of acetone on each 1 g of precipitate). Solutions resulting from the purification step were mixed together with the solution obtained from washing after elution and that from the first part of elution. Solid residue after purification was dried at 120–160 °C to constant mass of the aluminum perrhenate with a stoichiometric composition. Next thus was dried at 160–200 °C to obtain a constant mass of its anhydrous form. Solutions from mixing washings after elution and the first part of elution, were treated with active aluminum oxide in such amount to obtain up to 80 wt.% of rhenium in final precipitate (product B) and then directed to complete drying with intensive mixing at the temperature below 80 °C. Obtained precipitate that contained up to 80 wt.% of rhenium was sent to the calcination and activation. It should be mentioned that both materials (products A and B) are suitable substrates for rhenium catalyst production.

### 3.3. Hexamminecobalt(III) Perrhenate

Ammonia complexes of perrhenate are compounds of ML_x_(ReO_4_)_y_ type, containing coordinated ReO_4_^−^ ions, where M is either nickel or cobalt, and L is a coordinated NH_3_ group. A method of anhydrous hexaamminecobalt(III) perrhenate production based on reaction (1) with perrhenic acid and hexaamminecobalt(III) chloride, was described in this paper:

[Co(NH_3_)_6_]Cl_3_+3HReO_4_ → [Co(NH_3_)_6_](ReO_4_)_3_ + 3HCl (1)

Possible replacement of commercial product by obtained hexaamminecobalt(III) chloride and ammonia solution containing cobalt was checked. Moreover, the influence of selected factors on efficiency and composition of obtained [Co(NH_3_)_6_](ReO_4_)_3_·2H_2_O, i.e., hexaamminecobalt(III) chloride solution concentration, process temperature, reaction time, excess of perrhenic acid, and concentration of perrhenic acid, were investigated. The possibilities to replace perrhenic acid by other precipitating agents like ammonium perrhenate or anhydrous cobalt(II) perrhenate to produce [Co(NH_3_)_6_](ReO_4_)_3_·were investigated. The effect of hexaamminecobalt(III) perrhenate concentration were conducted based on the following procedure: 0.1 dm^3^ of 5.0–50.0 g/dm^3^ hexamminecobalt(III) chloride solution was magnetically mixed with aqueous solution of perrhenic acid (670.0 g/dm^3^ Re with 30 or 60% excess of rhenium to cobalt) at room temperature for half an hour. Phases were separated, and cobalt content in solution was analyzed, while the precipitate was analyzed with respect to the cobalt, ammonium and rhenium ions.

Effect of temperature was examined in the range from 10 to 80 °C. The reaction time was tested in the range from 0.5 to 5.0 h. The excess of rhenium was investigated as follows: 0.1 dm^3^ of 20.0 g/dm^3^ hexaamminecobalt(III) chloride solution was magnetically mixed with solution containing 569.0 g/dm^3^ of perrhenic acid, satisfying an excess of rhenium vs cobalt ranging from 10 to 200%. A trial with stochiometric amount of rhenium and cobalt was performed as well. Experiments were conducted at room temperature, intensively mixing with precipitate for 30 min.

The influence of perrhenic acid concentration on process efficiency was investigated as follows: 0.1 dm^3^ of 50.0 g/dm^3^ hexaamminecobalt(III) chloride solution was mixed with aqueous solution of perrhenic acid of concentration 107.0 to 508.0 g/dm^3^ Re with 60% excess of rhenium to cobalt, at room temperature intensively mixing with precipitate for 30 min.

The type of rhenium substrate was tested using the following procedure: 0.1 dm^3^ of 30.0 g/dm^3^ hexaamminecobalt(III) chloride solution was mixed with ammonium perrhenate either in solid state or as water solution, or with cobalt(II) perrhenate in solid state. Research was conducted at room temperature intensively mixing with precipitate for half an hour. An 60% excess of rhenium to cobalt was used. 

Experiments concerning possible substrate change were also an important part of the research. These were done by mixing 50.0 g of cobalt(II) chloride hexahydrate and 33.0 g of ammonium chloride in 0.3 dm^3^ of water. Then 10.0 g of activated carbon and 0.45 dm^3^ of ammonia solution were added. After cooling in ice bath, 0.04 dm^3^ of 30% hydrogen peroxide was carefully added drop by drop, to prevent the temperature from exceeding 10 °C. The resulting mixture was heated to 60 °C by 30 min and then cooled again in an ice bath. Crystallized product and activated carbon were separated from the solution, and dissolved in 0.4 dm^3^ of hot water with 0.01 dm^3^ of HCl. Next the mixture was heated and activated carbon was separated from the solution. The resulting solution was divided into two parts. The first part was used in the synthesis of hexaamminecobalt(III) perrhenate, while the second was placed in an ice bath to crystallize hexaamminecobalt(III) chloride. Crystallized product was separated from the filtrate, washed with water containing ice and then dried in the air. After drying (>120 °C) the content of chlorine, cobalt and ammonium ions, were analyzed. Crystallized product was dissolved in water to obtain hexaamminecobalt(III) chloride solution of 30.0 g/dm^3^ concentration. To 0.1 dm^3^ of such solution, 664 g/dm^3^ of Re as perrhenic acid solution was added with 30% excess of rhenium to cobalt. Trials were performed at room temperature with intensive mixing of the solution with precipitate for 30 min. Precipitate and filtrate were analyzed regarding cobalt content. Additionally in the case of precipitates the content of rhenium and ammonia was also analyzed. Similar test was performed for ammonia solution containing cobalt obtained directly after separation from activated carbon. The presented methodology was also performed on a 10-times larger scale.

### 3.4. Analytical Methods

The Department of Analytical Chemistry at IMN was responsible for all the necessary analysis. Rhenium content in product was analyzed using a gravimetric method with tetraphenylarsonium chloride (TPAC) as the precipitating agent, while aluminum and copper(II) were analyzed using flame atomic emission spectroscopy (FAES, spectrophotometer AAS novAA400, Persee, Auburn, AL, USA). Ammonia was analyzed with gravimetric method prior to NH_3_ distillation (error ± 0.05 g/dm^3^), while chlorine by potentiometric titration. Solutions were characterized with respect to Re, Co, Ni and Cr content by flame atomic absorption spectroscopy (FAAS, SOLAAR S4, THERMO, Waltham, MA, USA) equipped with flame module and deuterium background correction with measurement error ±0.2%. Analysis of most important contaminations i.e., Mo, Na, Ni, Co, Pb, was performed using ICP-MS (inductively coupled plasma mass spectrometry, ICP MS NexION, PerkinElmer, Waltham, MA, USA), while Ca, Fe, K, Mg, Zn by ICP-OES (inductively coupled plasma—optical emission spectrometer, ULTIMA 2, HORIBA Jobin-Ivon, Kyoto, Japan), and graphite furnace atomic absorption spectroscopy (GFAAS, Z-2000, HITACHI, Tokyo, Japan) with graphite cells Thermal stability analysis was performed using thermobalance HG63, Mettler Toledo (Columbus, OH, USA), at the temperature range from 60 to 200 °C. Thermal analysis was performed in a broader range from 30 to 1000 °C, at heating rate 10 °C/min, in an Ar atmosphere with a flow rate 150 cm^3^/min using a Netzsch STA 409 C/CD with DTA/TG (Selb, Germany). X-ray powder diffraction analysis was carried out using Co Kα radiation in 2θ range 10°–100° (XRD 7, Seifert-FPM, Freiberg, Germany).

## 4. Conclusions

It was determined that aluminum and copper(II) perrhenates of high purity may be produced by the ion-exchange method using the strongly acidic cation-exchange resins PFC 100x10 and C160, respectively. The developed technologies produce high purity perrhenates of the following compositions: in case of Al(ReO_4_)_3_–71.85% Re, 3.48% Al and <5 ppm Ni; 8 ppm Co; 15 ppm Ca; 10 ppm K; <2 ppm Mg; 15 ppm Na; <5 ppm Pb <5 ppm Mo and 10 ppm Zn, while for Cu(ReO_4_)_2_–66.02% Re, 11.27% Cu and <10 ppm Ni; <5 ppm Co; <3 ppm Ca; <10 ppm K; <2 ppm Mg; <10 ppm Na; <5 ppm Pb <5 ppm Mo and <3 ppm Zn. In (ReO_4_)_3_ technology the product B, which was Re adsorbed on Al_2_O_3_ from 73.2% to 79.6% being the proper heterogeneous catalyst precursor, was additionally produced. The produced [Co(NH_3_)_6_](ReO_4_)_3_ contained 61.3% Re, 6.5% Co, 11.2% NH_3_, <20 ppm Cl, <5 ppm Na, <5 ppm Ca, <5 ppm Mg, <5 ppm Pb, <5 ppm Fe, <5 ppm Mo, <5 ppm Cr, <5 ppm Ni, <10 ppm K, <3 ppm Zn. All developed technologies are practically waste-free processes thanks to a proper recycling of spent solutions. Metal losses, i.e., Al, Cu, Re and Co for each of the developed technology were limited to a minimum and did not exceed 0.1%. The purity of produced compounds may enable their use in catalysis.

## Figures and Tables

**Table 1:** Results of copper(II) ion sorption with C160 ionite–ten cycles.

Cycles	Volume of Solution After Sorption, dm^3^	Cu Concentration in Solution after Sorption, g/dm^3^	Volume of Washings after Sorption, dm^3^	Cu Concentration in Washings, g/dm^3^	Cu Loss, %	Cu Sorption Efficiency, %	Cu Saturation Degree, %
**I**	8.5	0.04	1.0	0.01	0.35	99.13	3.97
**II**	8.5	0.05	0.9	0.02	0.44	98.89	3.96
**III**	8.5	0.05	1.1	0.02	0.45	98.88	3.96
**IV**	8.4	0.07	1.6	0.03	0.64	98.41	3.94
**V**	8.2	0.08	1.3	0.04	0.71	98.23	3.93
**VI**	8.5	0.10	1.0	0.08	0.93	97.68	3.91
**VII**	8.1	0.56	1.4	0.10	4.68	88.31	3.53
**VIII**	8.1	1.10	2.4	0.30	9.63	75.93	3.04
**IX**	8.4	2.50	1.1	1.10	22.21	44.48	1.78
**X**	8.0	2.60	1.5	1.50	23.05	42.38	1.70

**Table 2:** Results of copper(II) ions sorption with C160 ionite–five cycles (after regeneration).

Cycles	Volume of Solution After Sorption, dm^3^	Cu Concentration in Solution after Sorption, g/dm^3^	Volume of Washings after Sorption, dm^3^	Cu Concentration in Washings, g/dm^3^	Cu Loss, %	Cu Sorption Efficiency, %	Cu Saturation Degree, %
**I**	8.4	0.03	1.1	0.01	0.26	99.34	3.97
**II**	8.2	0.03	1.3	0.02	0.27	99.32	3.97
**III**	8.4	0.04	1.2	0.03	0.37	99.07	3.96
**IV**	8.1	0.05	1.4	0.02	0.43	98.92	3.96
**V**	8.0	0.08	1.5	0.04	0.70	98.25	3.93

**Table 3:** Results of copper(II) ions elution–ten cycles.

Cycles	Volume of Solution for Neutralization, dm^3^	Concentration in Neutralization Solution, g/dm^3^		Volume of First Part of Solution after Elution *, dm^3^	Concentration in Recycled Solution, g/dm^3^		Cu Elution Efficiency, %	Mass of Cu Remaining in Ionite, g
		Cu	Re		Cu	Re		
**I**	5.65	6.77	42.03	1.00	0.50	2.00	96.47	0.90
**II**	5.25	7.40	45.52	0.90	0.90	1.80	95.97	0.82
**III**	4.75	8.12	49.86	1.30	0.80	2.50	95.54	0.76
**IV**	5.35	7.16	44.85	1.10	1.50	2.20	95.50	0.15
**V**	5.15	7.25	45.96	1.10	1.10	2.10	94.67	0.89
**VI**	5.35	6.82	44.56	0.90	1.80	2.50	91.31	1.85
**VII**	5.05	6.49	47.35	1.00	3.20	2.50	88.16	1.20
**VIII**	4.65	5.33	51.24	1.50	4.50	2.60	78.52	0.03
**IX**	5.05	1.39	42.66	1.60	3.00	2.60	39.42	5.99
**X**	5.15	1.40	46.22	1.60	2.60	2.60	31.51	11.56

* solution recycled to eluting agent preparation step.

**Table 4:** Results of copper(II) ions elutions–five cycles (after regeneration).

Cycles	Volume of Solution for Neutralization, dm^3^	Concentration in Neutralization Solution g/dm^3^		Volume of First Part of Solution after Elution *, dm^3^	Concentration in Recycled Solution, g/dm^3^		Cu Elution Efficiency, %	Mass of Cu Remaining in Ionite, g
		Cu	Re		Cu	Re		
**I**	5.65	6.98	42.03	1.00	0.10	2.00	99.34	0.19
**II**	5.25	7.48	45.52	0.90	0.60	1.80	98.33	0.12
**III**	5.05	7.73	47.49	1.00	0.50	1.70	98.11	0.22
**IV**	5.15	7.26	48.20	1.00	1.50	2.20	93.96	0.91
**V**	5.05	7.43	46.56	1.10	1.30	2.10	93.28	1.27

* solution recirculated to eluting agent preparation step.

**Table 5:** Neutralization and concentration results.

Cycles	Volume of Solution for Neutralization, dm^3^	Concentration in Solution for Neutralization, g/dm^3^		Neutralizing Agent/Mass of Agent/Temperature	pH	Neutralization Time, h	Mass of Copper(II) Perrhenate, %	Re Loss %
		Cu	Re					
**I**	5.65	6.77	42.03	CuO, 2.9 g, 20 °C	4.3	5.0	358.2	0.42
**II**	5.25	7.40	45.52	Cu(OH)_2_, 3.0 g, 20 °C	5.6	3.0	360.4	0.44
**III**	4.75	8.12	49.86	CuO, 2.3 g, 40 °C	4.5	4.0	357.5	0.35
**IV**	5.35	7.16	44.85	Cu(OH)_2_, 4.1 g, 40 °C	5.8	2.5	360.7	0.76
**V**	5.15	7.25	45.96	CuO, 3.8 g, 80 °C	4.7	2.0	357.5	0.29
**VI**	5.35	6.82	44.56	Cu(OH)_2_, 6.5 g, 80 °C	6.1	1.5	360.3	0.23
II *	5.25	7.48	45.52	CuO, 1.9 g, 80 °C	4.8	1.5	360.6	0.39
III *	5.05	7.73	47.49	Cu(OH)_2_, 2.9 g, 80 °C	5.9	1.5	361.6	0.46

* cycles after regeneration.

**Table 6:** Composition of obtained copper(II) perrhenate.

Copper(II) Perrhenate	Re%	Cu%	Ni ppm	Co ppm	Mg ppm	Ca ppm	Na ppm	Zn ppm	Mo ppm	K ppm	Pb ppm
**Crude perrhenate from cycle V**	65.99	11.23	50	56	<2	10	34	3	5	13	<5
**Precipitate after stage I of purification (from cycle V)**	66.00	11.27	15	12		5	15	<3	<5	10	<5
**Precipitate after stage II of purification (from cycle V)**	66.02	11.27	<10	<5		<3	<10	<3	<5	<10	<5
**Crude from cycle VI**	65.98	11.24	45	55		12	32	<3	5	12	<5
**Precipitate after stage I of purification (from cycle VI)**	66.01	11.26	20	10		4	12	<3	<5	10	<5
**Precipitate after the II stage of purification (from cycle VI)**	66.02	11.27	<10	<5		<3	<10	<3	<5	<10	<5
Crude perrhenate from cycle I *	65.99	11.24	30	23		10	20	3	5	13	<5
Crude perrhenate from cycle III *	66.00	11.23	52	58		14	33	6	7	18	<5

* cycles after regeneration, ** perrhenate after drying 140 °C.

**Table 7:** The results of the aluminum ion sorption process, using PFC 100 × 10 ionite–ten cycles.

Cycles	Volume of Solution after Sorption, dm^3^	Concentration of Al in Solution after Sorption, g/dm^3^	Volume of Washings after Sorption, dm^3^	Concentration of Al in Washings, g/dm^3^	Aluminum Sorption Efficiency, %	Saturation Degree of Ionite by Al^3+^, %
**I**	6.5	0.008	1.00	0.100	99.79	2.99
**II**	8.1	0.008	0.90	0.100	99.75	3.00
**III**	8.1	0.090	2.40	0.050	97.17	2.92
**IV**	8.1	0.150	3.90	0.100	99.48	2.92
**V**	8.1	0.200	5.40	0.150	92.31	2.92
**VI**	9.0	0.200	5.50	0.160	91.52	2.89
**VII**	8.5	0.230	7.00	0.200	89.45	2.84
**VIII**	8.5	0.700	8.00	0.500	68.86	2.20
**IX**	8.5	2.000	9.00	1.500	23.65	0.95
**X**	8.5	3.400	10.00	2.500	10.91	0.66

**Table 8:** The results of the aluminum ion sorption process, using PFC 100 × 10 ionite–five cycles (after regeneration).

Cycles	Volume of Solution after Sorption, dm^3^	Concentration of Al in Solution after Sorption, g/dm^3^	The Volume of Washings after Sorption, dm^3^	Concentration of Al in Washings, g/dm^3^	Aluminum Sorption Efficiency, %	Saturation Degree of Ionite by Al^3+^, %
**I**	6.5	0.009	1.0	0.010	99.77	2.99
**II**	8.1	0.009	0.9	0.010	99.73	3.00
**III**	8.1	0.090	2.4	0.050	99.17	2.92
**IV**	8.1	0.140	3.9	0.050	95.69	2.93
**V**	8.1	0.150	5.4	0.140	93.54	2.95

**Table 9:** The results of the elution of aluminum ions–ten cycles.

Cycles	Volume of Solution for Product A Precipitation, dm^3^	Concentration in Solution for Product A Precipitation, g/dm^3^		Volume of Solution for Product B Precipitation, dm^3^	Concentration in Solution for Product B Precipitation, g/dm^3^		Aluminium Elution Efficiency, %	Amount of Al for Product A Extraction, %	
		Al	Re		Al	Re		Al	Re
**I**	3.0	7.30	189.00	3.30	2.40	18.97	99.61	73.05	26.56
**II**	2.6	8.90	209.00	3.65	1.73	23.63	98.27	76.79	21.48
**III**	3.2	7.60	175.00	2.70	1.57	19.68	97.94	83.09	14.85
**IV**	3.0	7.80	187.00	2.90	1.62	19.90	96.05	79.36	16.70
**V**	3.0	7.70	187.00	2.90	1.62	19.90	95.25	78.38	16.88
**VI**	2.8	7.30	179.00	3.10	2.26	20.45	94.82	69.31	25.51
**VII**	2.5	7.80	205.00	2.70	2.61	27.93	93.30	66.76	26.53
**VIII**	2.0	5.70	205.00	2.90	3.03	28.21	91.71	48.20	43.51
**IX**	2.0	2.20	201.00	3.40	0.92	22.71	79.79	38.15	41.64
**X**	2.0	0.90	167.00	2.40	0.62	17.50	49.70	4.58	45.12

**Table 10:** The results of the elution of aluminum ions–five cycles (after regeneration).

Cycles	Volume of Solution for Product A Precipitation, dm^3^	Concentration in Solution for Product A Precipitation, g/dm^3^		Volume of Solution for Product B Precipitation, dm^3^	Concentration in Solution for Product B Precipitation, g/dm^3^		Aluminium Elution Efficiency, %	Amount of Al for Product A Extraction A, %	
		Al	Re		Al	Re		Al	Re
**I**	3.0	7.10	189.00	3.45	2.44	19.10	99.33	70.97	28.36
**II**	3.0	7.90	180.00	3.45	1.71	23.55	98.68	78.71	19.91
**III**	3.0	7.90	182.00	2.90	1.61	19.90	97.33	80.84	16.49
**IV**	3.1	7.80	178.00	2.80	1.61	19.79	97.09	81.46	15.63
**V**	3.0	8.10	187.00	2.90	1.62	19.90	98.93	82.75	16.18

**Table 11:** Neutralization and concentration results–product A.

Cycles	Volume of Solution for Neutralization, dm^3^	Concentration in Solution for Neutralization, g/dm^3^		Neutralizing Agent/Mass of Agent/Temperature	pH	Neutralization Time, h	Mass of Aluminium Perrhenate, %	Efficiency, %
		Al	Re					
**I**	3.0	7.30	189.00	Al(OH)_3_, 16.0 g, 20 °C	3.5	5.0	788.52	99.87
**II**	2.6	8.90	209.00	Al(OH)_3_, 9.10 g, 40 °C	3.8	4.0	754.34	99.69
**III**	3.2	7.60	175.00	Al(OH)_3_, 8.00 g, 60 °C	4.2	3.0	777.00	99.64
**IV**	3.0	7.80	187.00	Al(OH)_3_, 10.80 g, 80 °C	4.2	1.5	776.11	99.35
**VIII**	2.0	5.70	205.00	Al(OH)_3_, 24.40 g, 80 °C	4.3	1.5	540.23	94.63

**Table 12:** Neutralization and concentration results–product B.

Cycles	Volume of Solution for Neutralization, dm^3^	Concentration in Solution for Neutralization, g/dm^3^		Neutralizing Agent/Temperature/Mass of Agent	Mass of Precipitate, %	Re Concentration %
		Al	Re			
**I**	3.30	2.40	18.97	Al_2_O_3_ −20 °C–60 g	86.32	72.5
**II**	3.65	1.73	23.63	Al_2_O_3_ −40 °C–60 g	108.36	79.6
**III**	2.70	1.57	19.68	Al_2_O_3_ −60 °C–60 g	73.18	72.6
**IV**	2.90	1.62	19.90	Al_2_O_3_ −80 °C–60 g	78.21	73.8
**VIII**	2.90	3.03	28.21	Al_2_O_3_ −80 °C–80 g	111.70	73.2

**Table 13:** Composition of obtained aluminum perrhenate.

Aluminium Perrhenate	Re%	Al%	Ni ppm	Co ppm	Mg ppm	Ca ppm	Na ppm	Zn ppm	Mo ppm	K ppm	Pb ppm
**Crude perrhenate from cycle V**	60.59	2.93	8	10	<2	20	34	13	5	13	<5
Crude perrhenate * after stage I of purification (from cycle IV)	71.85	3.48	<5	8	<2	15	15	10	<5	10	<5
Crude perrhenate * after stage of the II purification (from cycle IV)	71.85	3.48	<5	<5	<2	<3	<10	<3	<5	<10	<5

** perrhenate after drying at 200 °C.

**Table 14:** Results of the temperature effect on the efficiency and composition of the hexaamminecobalt(III) perrhenate.

Temperature, °C	Volume of Solution after Precipitation, dm^3^	Concentration of Cobalt in Solution after Precipitation, g/dm^3^	Mass of Precipitate, g	Composition of Precipitate, %			Efficiency of Precipitation, %
				Re	NH_3_	Co	
10	0.110	0.23	9.7	58.8	10.9	5.9	91.2
20	0.110	0.19	10.2	58.9	10.7	6.1	95.9
40	0.105	0.18	10.0	58.7	10.9	6.2	94.0
60	0.100	0.30	9.9	58.8	10.8	5.8	93.1
80	0.650	0.28	9.8	58.9	10.9	5.9	92.1

**Table 15:** Results of the reaction time on the efficiency and composition of the hexaamminecobalt(III) perrhenate.

Reaction Time, min	Volume of Solution after Precipitation, dm^3^	Concentration of Cobalt in Solution after Precipitation, g/dm^3^	Mass of Precipitate, g	Composition of Precipitate, %			Efficiency of Precipitation, %
				Re	NH_3_	Co	
30	0.110	0.13	9.7	58.8	10.9	6.2	91.2
60	0.110	0.19	10.2	58.9	10.7	6.3	95.9
180	0.105	0.23	10.0	58.9	10.9	6.2	94.0
300	0.105	0.14	9.9	58.8	10.8	6.2	93.1

**Table 16:** Results of the effect of perrhenic acid concentration on the efficiency and composition of hexaamminecobalt(III) perrhenate dihydrate.

Concentration of Re, g/dm^3^	The volume of Solution after Precipitation, dm^3^	Concentration of Cobalt in Solution after Precipitation, g/dm^3^	Mass of Precipitate, g	Composition of Precipitate, %			Efficiency of Precipitation, %
				Re	NH_3_	Co	
16	0.220	4.7	8.7	58.9	10.7	6.3	49.1
31	0.223	0.0012	13.0	58.9	10.8	6.1	73.3
63	0.185	0.0014	15.6	58.7	10.9	6.2	88.0
127	0.168	0.0014	15.8	58.8	10.8	6.2	89.1
254	0.140	0.0016	16.2	58.9	10.6	6.1	91.4
508	0.108	0.0017	16.0	58.9	10.7	6.2	90.3

**Table 17:** Results of the effect of changing the rhenium substrate on the efficiency and composition of hexaamminecobalt(III) perrhenate.

Rhenium Substrate	Cobalt Substrate	Composition of Precipitate, %			Efficiency of Precipitation, %
		Re	NH_3_	Co	
HReO_4_	[Co(NH_3_)_6_]Cl_3_	58.9	10.7	6.2	90.3
NH_4_ReO_4_		64.8	7.6	3.2	-
Co(ReO_4_)_2_		56.8	8.7	8.0	-

**Table 18:** Results of the effect of cobalt substrate change on the efficiency and composition of [Co(NH_3_)_6_](ReO_4_)_3_·2H_2_O.

Rhenium Substrate	Cobalt Substrate	Composition of Precipitate, %			Efficiency of Precipitation, %
		Re	NH_3_	Co	
HReO_4_	Commercial [Co(NH_3_)_6_]Cl_3_ synthesized [Co(NH_3_)_6_]Cl_3_ Co-containing ammonia solutions	58.8	10.8	6.3	90.2
		58.8	10.8	6.3	95.7
		58.9	10.7	6.2	96.0
